# Hsp70 May Be a Molecular Regulator of Schistosome Host Invasion

**DOI:** 10.1371/journal.pntd.0004986

**Published:** 2016-09-09

**Authors:** Kenji Ishida, Emmitt R. Jolly

**Affiliations:** 1 Department of Biology, Case Western Reserve University, Cleveland, Ohio, United States of America; 2 Center for Global Health and Diseases, Case Western Reserve University, Cleveland, Ohio, United States of America; George Washington University School of Medicine and Health Sciences, UNITED STATES

## Abstract

Schistosomiasis is a debilitating disease that affects over 240 million people worldwide and is considered the most important neglected tropical disease following malaria. Free-swimming freshwater cercariae, one of the six morphologically distinct schistosome life stages, infect humans by directly penetrating through the skin. Cercariae identify and seek the host by sensing chemicals released from human skin. When they reach the host, they burrow into the skin with the help of proteases and other contents released from their acetabular glands and transform into schistosomula, the subsequent larval worm stage upon skin infection. Relative to host invasion, studies have primarily focused on the nature of the acetabular gland secretions, immune response of the host upon exposure to cercariae, and cercaria-schistosomulum transformation methods. However, the molecular signaling pathways involved from host-seeking through the decision to penetrate skin are not well understood. We recently observed that heat shock factor 1 (Hsf1) is localized to the acetabular glands of infectious schistosome cercariae, prompting us to investigate a potential role for heat shock proteins (HSPs) in cercarial invasion. In this study, we report that cercarial invasion behavior, similar to the behavior of cercariae exposed to human skin lipid, is regulated through an Hsp70-dependent process, which we show by using chemical agents that target Hsp70. The observation that biologically active protein activity modulators can elicit a direct and clear behavioral change in parasitic schistosome larvae is itself interesting and has not been previously observed. This finding suggests a novel role for Hsp70 to act as a switch in the cercaria-schistosomulum transformation, and it allows us to begin elucidating the pathways associated with cercarial host invasion. In addition, because the Hsp70 protein and its structure/function is highly conserved, the model that Hsp70 acts as a behavior transitional switch could be relevant to other parasites that also undergo an invasion process and can apply more broadly to other organisms during morphological transitions. Finally, it points to a new function for HSPs in parasite/host interactions.

## Author Summary

Parasitic schistosome worms cause morbid disease in over 240 million individuals worldwide. Acute infections with these worms can lead to Katayama fever, while chronic infections can lead to portal hypertension, enlarged abdomen, and liver damage. The infective larval stage, called cercariae, are free-swimming and can detect, seek, and penetrate human skin to enter the human host circulatory system, eventually developing into egg-laying adult worms that cause schistosomiasis. Molecular pathways associated with the initial cercarial invasion of the host, however, are largely unknown, especially with respect to the parasite-specific signals involved in host detection and subsequent decision to invade. Here, we describe a role for Hsp70 in cercarial invasion behavior. To date, only generic stimulation with skin lipid, linoleic acid or L-arginine are known to induce cercarial invasion behavior; thus, we can begin an initial investigation of molecular requirements for host invasion and environment transition for schistosomes and possibly other parasitic organisms.

## Introduction

Schistosome parasites have six different morphological stages during their life cycle, which requires an intermediate molluscan and a definitive mammalian host that the parasite must correctly identify and invade. Free-swimming, freshwater cercariae (singular: cercaria) are released from infected molluscs and invade mammals and humans for further development into larval worms called schistosomula (singular: schistosomulum or schistosomule). Schistosomula adapt to survival in the host blood environment, evade the immune system, develop a gut to begin digesting red blood cells, elongate and traverse the human circulatory system, and eventually develop into egg-laying adult worms [1].

Cercariae are highly adapted for swimming and invading their mammalian hosts. Transcriptional studies show that cercariae have elevated expression of genes associated with metabolism and motility when compared with other stages [2, 3]. Free-swimming cercariae have a limited energy supply and a limited duration during which they can infect their host [4]. Thus, they must correctly identify and quickly respond to an appropriate host (or source of chemoattractant), swim toward it, and begin the host penetration process. For the purposes of this report, we call this behavior cercarial honing or simply, honing. Swimming cercariae respond to changes in light levels, to thermal gradients, and to chemicals such as linoleic acid and L-arginine released from human skin [5–9]. After reaching the skin, the cercariae crawl along the skin surface until they identify a suitable location to penetrate. Parasite invasion through the skin involves the physical motion of swimming into the skin, in coordination with release of their acetabular gland contents, which include mucins to enhance the attachment to skin and proteases to degrade skin molecules [10–12].

While the ultrastructure of cercariae has been described before and after entry into the host [13–15], protein regulators of cercarial honing and invasion have not been studied, with the exception of two reports [16, 17]. In 1991, Matsumura and others proposed that protein kinase C and calcium metabolism are involved in proteolytic enzyme release from cercariae acetabular glands [16]. Almost 25 years later, Ressurreição followed up on the work by Matsumura and recently reported that PKC, ERK, and p38 MAPK phosphorylation is involved in release of proteolytic enzymes from cercarial acetabular glands following the observation that inhibition of PKC, ERK, and p38 MAPK activities blocked linoleic acid-induced release of acetabular gland contents [17]. The current report further explores the molecular requirements for cercarial host invasion. We identify heat shock protein 70 (Hsp70) as a potential molecular component involved in cercarial honing and show that inhibition of Hsp70 can bypass the requirement for linoleic acid, L-arginine, or any host-derived signal to induce cercarial host targeting behavior. Interestingly, numerous reports corroborate regulatory interplay between Hsp70, PKC, ERK, and p38 MAPK activities [18–21].

Several studies led us to investigate the potential role for a heat shock pathway during cercarial honing and invasion. First, the heat shock response has traditionally been associated with cellular stress [22–24], and cercariae are no exception to this, since they must transition from a cooler, low-saline and freshwater environment to the warmer, saline environment of a human host. Second, we recently observed an unexpected localization of heat shock factor 1 (Hsf1), the major transcriptional activator responsible for transcribing heat shock genes (such as *HSP70* and *HSP90*), to the acetabular glands of cercariae [25]. This observation helps corroborate the findings of another study that showed the presence of Hsp70 in released acetabular gland contents [26]. Third, the heat shock response may play a role in other stages of schistosome infection as well. In particular, an induced heat shock response in the schistosome intermediate host *Biomphalaria glabrata* renders them susceptible to schistosome infection, while absence of a strong heat shock response leads to resistance [27]. Together, these studies suggest an important role for a heat shock pathway in parasitic schistosomes.

Hsp70, a member of the heat shock protein (HSP) superfamily, is structurally and evolutionarily conserved from prokaryotes to eukaryotes and generally functions as a chaperone protein that aids in (re)folding nascent and denatured proteins through interactions with its substrate domain and ATP hydrolysis (for review, [28]). However, additional roles for Hsp70 outside of its well-established chaperone functions have also been described. Together with various co-chaperones, Hsp70 can also direct signaling pathways that control cell death, differentiation, homeostasis, and proliferation by modulating the function of key regulatory proteins (client proteins) [29]. This is observed in the regulation of tumor necrosis factor receptor 1 (TNFR1) signaling [30]. Aggregation of TNFR1 leads to cell death; however, TNFR1 aggregation is inhibited when TNFR1 interacts with silencer of death domain (SODD). Hsp70 is thought to bind to SODD, modifying it to induce SODD/TNFR1 interaction, thereby inhibiting TNFR1-dependent cell death [30]. Hsp70 also plays a role in modulating Smad-mediated transcription [31]. Smad proteins are essential transducers of the transforming growth factor superfamily. Smad-mediated transcription is enhanced by the activity of the melanocyte specific gene (Msg1) protein, a transcriptional activator that cannot independently bind DNA but does so indirectly through interaction with p300/CBP. Hsp70 forms a complex with Msg1, suppressing its interaction with p300/CBP, and consequently blocks Msg1 enhancement of Smad-mediated transcription [31]. As another example, in clathrin-mediated endocytosis, Hsp70 binds and holds clathrin triskelia, preventing their aggregation during the uncoating of clathrin-coated vesicles; in the other half of the clathrin cycle, Hsp70 releases the triskelia to allow the coating of new vesicles upon activation by some unknown signal(s) [32]. The role of Hsp70 in clathrin-mediated endocytosis resembles that which we propose here for cercarial honing, especially with respect to the sequestering of important cellular components until Hsp70 receives an activating signal to release its client protein. While identification of the mechanism for Hsp70 mediated regulation for clathrin-mediated endocytosis is a topic of much interest [33–35], a similar mechanism and question just as interesting may apply to the cercarial honing and invasion process.

In this study, we treated cercariae with modulators of Hsp70 protein that inhibit or activate Hsp70 via different mechanisms to explore whether Hsp70 functions in cercarial host invasion. Of interest, we found that 2-phenylethynesulfonamide (PES), also known as pifithrin-μ, initiated the process of cercarial honing and invasion in the absence of any host-specific stimulants such as skin lipids or linoleic acid, and it did so with 100% effectivity, which is greater than that observed with either skin lipids or linoleic acid, albeit at a slower rate. PES specifically binds to Hsp70 (K_d_ ~ 2.9 μM), and its derivatives do not interact with Grp75 or Grp78, organelle-specific members of the Hsp70 family [36, 37]. X-ray crystallographic analysis shows that PES interacts with residues L394, P398, L401, G484, N505, and D506 in human Hsp70. We propose a model that Hsp70 is involved in a signaling pathway that causes cercariae to begin host invasion maneuvers and that inhibition of Hsp70 bypasses the need for upstream host signals that normally initiate this process.

We have recorded and observed over 200 videos of cercarial mobility in response to small molecule modulators that target Hsp70, heat shock protein 90 (Hsp90), or apoptosis. To our knowledge, this is the first investigation of a molecular signaling pathway in cercariae that points to a role for Hsp70 as a regulatory factor for the transition between parasite development stages. In addition to providing a potential pathway to which we can direct drug development against schistosomes, these data could apply more broadly to other parasites and to other organisms during transitions or periods of rapid development [38]. Finally, we add to the current model in describing cercarial host invasion.

## Methods

### Phylogenetic analysis of Hsp70

Protein sequences of Hsp70 from various species most closely related to that of *Schistosoma mansoni* (NCBI accession numbers: CCD76164 (Smp_106930) and CCD76236 (Smp_049550) were identified by the NCBI BLASTp function [39] and aligned using ClustalW2 using its default parameters [40]. A phylogenetic tree was generated using the output of the ClustalW2 alignment and TreeView X software.

### Animals and parasites

*Biomphalaria glabrata* snails infected with *S*. *mansoni* (NMRI strain) were obtained from Biomedical Research Institute (BRI; Rockville, MD). Cercariae were collected from infected snails by light-induced shedding: the snails were kept in the dark overnight and then placed under bright light for 2 hours [41].

### Parasite observation and treatments

Cercariae were observed in 12-well or 24-well culture plates (respectively about 1,000 or 500 cercariae per well) using an inverted (VanGuard 1493INi) and upright stereo (Olympus SZ30) microscope fitted with a camera (Canon T5i). Videos were captured with the focus on the bottom of the wells at 40× and 10× magnification and a camera setting of 1280 by 720 at 60 fps. Images shown in figures are frames extracted from the videos.

Treatments of cercariae included the addition of the following substances; the treatment concentrations were chosen based on those used in the studies indicated (typically increased several-fold over those used in cell-based studies): human skin lipid (finger swipe), linoleic acid (Sigma L1012) [42], Hsp70 modulators 2-phenylethynesulfonamide (PES; Sigma P0122) [36], MKT-077 (Sigma M5449) [43], 115-7c (Stressmarq SIH-123) [44], and VER-155008 (Sigma SML0271) [45], Hsp90 inhibitors geldanamycin and 17-dimethylaminoethylamino-17-demethoxygeldanamycin (17-DMAG; these Hsp90 inhibitors were a kind gift of Giselle Knudsen and Jonathan Choy from the Small Molecule Discovery Center at UCSF) [46], pan-caspase inhibitor Z-VAD-FMK (Santa Cruz Biotechnologies sc-3067) [47], anthelmintic praziquantel (Sigma P4668) [48], and adenosine phosphates ATP (Sigma A1852), AMP-PNP (non-hydrolyzable ATP analog; Sigma A2647), and ADP (Sigma A2754) [49, 50]. These substances were either vortexed with a volume of water before treatment or added directly to water containing cercariae. Cercariae were treated within 3 hours of collection, and the time points expressed in this report refer to the time elapsed after the administration of a given treatment.

## Results

### Schistosome Hsp70 is highly conserved

We obtained a 637 amino acid protein sequence for *S*. *mansoni* Hsp70 from NCBI (CCD76164) and used this sequence as a query (NCBI BLASTp) to identify homologous Hsp70 proteins from different organisms. Using the available sequences (incomplete sequences were omitted), we performed an alignment using ClustalW2 to determine the phylogenetic relationship among these proteins (S1 Fig). As expected, we found that *Sm*Hsp70 proteins are highly conserved across organisms with greater than 50% identity and that they cluster into different Hsp70 classes [51]. *Sm*Hsp70 (NCBI accession CCD76164, 637 amino acids (aa)) clustered with the human Hsp70 (NCBI accession NP_006588, 646 aa), which is constitutively expressed and recognized as the heat shock protein 70 cognate (Hsc70) protein. The second *Sm*Hsp70 protein (NCBI accession CCD76236, 648 aa) represents a non-constitutive heat-inducible form of Hsp70, and it clustered with *Hs*Hsp70 (NCBI accession AAI12964, 655 aa), also called heat shock protein 70 family A (Hsp70) member 5, which is localized to the lumen of the endoplasmic recticulum (ER) where it is thought to mediate protein trafficking of ER-derived proteins, thereby regulating protein signaling [52].

### Establishment of cercarial swimming in culture

Previously, we published the observation that *Sm*Hsf protein is localized to the acetabular glands of schistosome cercariae [25]. *Sm*Hsf is a transcriptional activator of HSPs. While we do not think that Hsf1 can directly regulate the actions of its transcriptional targets in acetabular glands, we became interested in the idea that Hsf1 or HSPs may be involved in the transition between cercariae and schistosomula, either for cercarial invasion or for newly transformed schistosomula.

**Fig 1:**
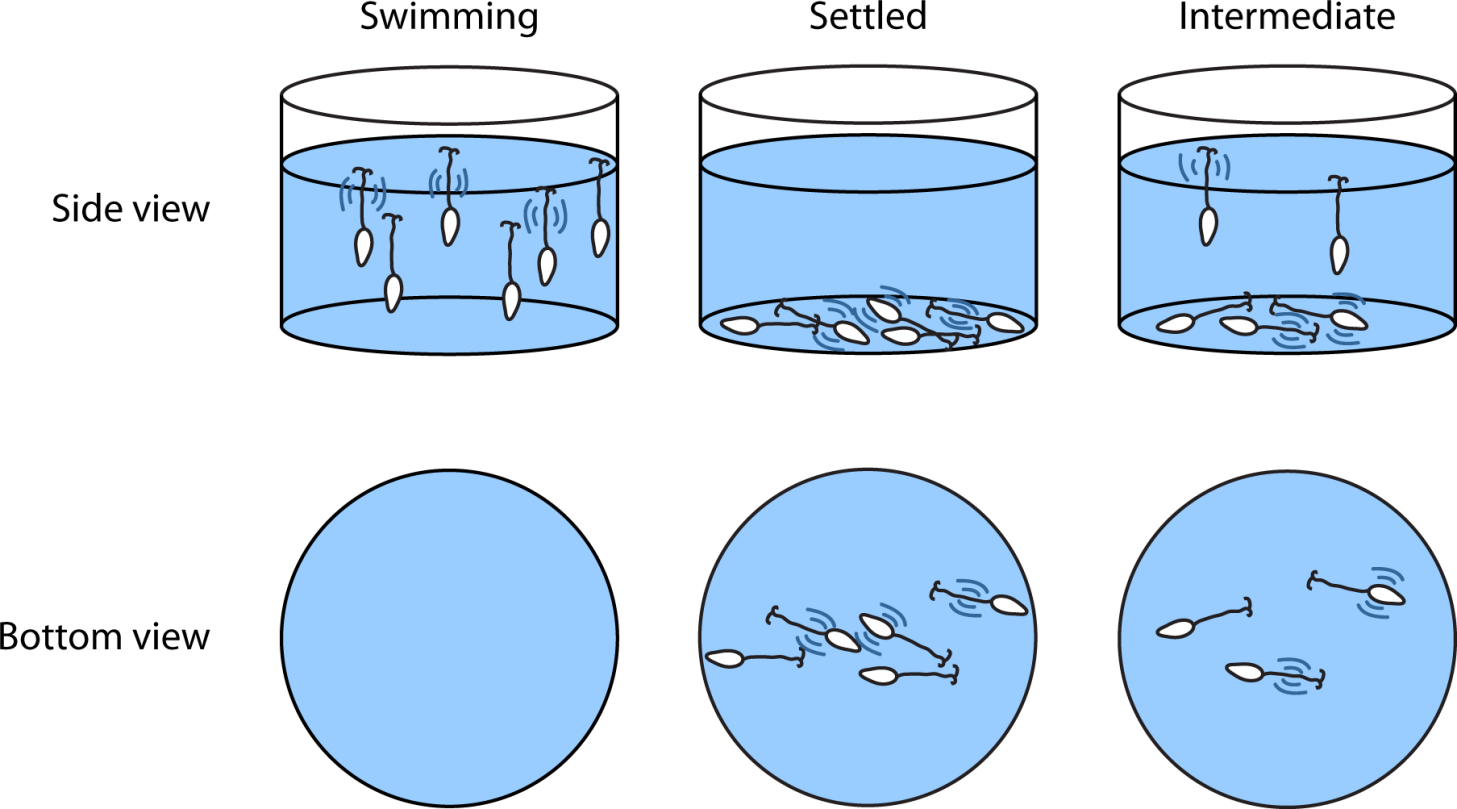
Visual illustration of cercariae swimming. During active swimming, most cercariae are found in the water column, while almost no cercariae can be seen at the bottom of the culture well. As cercariae hone, more of them can be seen at the bottom of the well when observed at higher magnification.

We began by experimentally repeating observations of cercarial responses to human skin lipid that have been well established since the 1970s [26, 53]. Our descriptions of cercariae are based on observations from inverted and upright microscopes. However, because cercariae continuously moved vertically in our 1 mL water samples, a consistent location to image between samples was not possible. Thus, images described here focus on the bottom of the culture wells, with approximately 1,000 cercariae per well for a 12-well culture plate or 500 cercariae per well for a 24-well culture plate. When observing cercariae by microscopy in a culture well, the relatively large depth of the water column and the nature of standard microscopes precludes a meaningful side-view visualization. Swimming cercariae, in wait of a host, are distributed vertically in a water column with few touching the bottom surface of a culture well. Thus, most cercariae will not be seen at the bottom of a culture well from this viewpoint. In contrast, when the cercariae have settled in response to a stimulus, many more cercariae can be observed at the bottom of a culture well (Fig 1). The apparent lack of cercariae in some of the images described later is not caused by a discrepancy in the number of cercariae added, but rather by their specific distribution (vertical and horizontal) in the water column.

Since many drugs are often diluted or dissolved in DMSO, we established a baseline for cercarial DMSO tolerance, relative to what we observed in water. We compared cercariae treated with filtered water, 0.5% DMSO, and 1% DMSO. Cercariae treated with water and 0.5% DMSO were distributed in a similar manner and exhibited a similar swim (up)-sink-swim behavior at both 10 minutes and 2 hours (S1 Video).

### Treatment of cercariae with human skin lipid, linoleic acid, and PES

**Fig 2:**
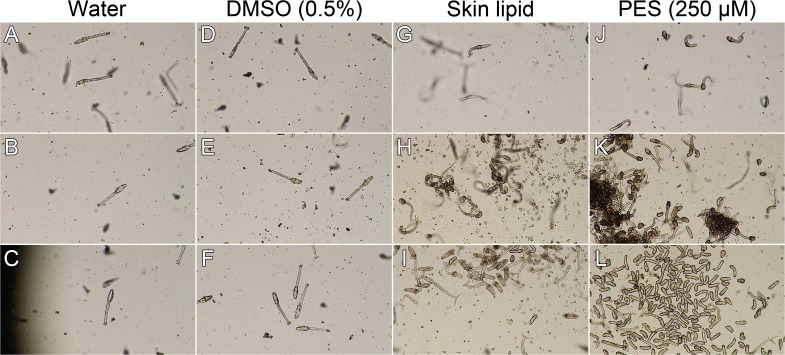
Cercariae treated with PES hone and transform more completely than those treated with skin lipid. Cercariae were treated with filtered water (A-C), 0.5% DMSO (D-F), human skin lipid (G-I), or 250 μM PES (J-L), and observed at various time points. The treatments and observation timings are as follows: (A) water, 8 minutes; (B) water, 56 minutes; (C) water, 1 hour 45 minutes; (D) DMSO, 9 minutes; (E) DMSO, 57 minutes; (F) DMSO, 1 hour 46 minutes; (G) lipid, 0 minutes; (H) lipid, 9 minutes; (I) lipid, 1 hour; (J) PES, 3 minutes; (K) PES, 51 minutes; (L) PES, 1 hour 42 minutes. Each treatment used about 1,000 cercariae in a volume of 1 mL in a 12-well plate well (40× view).

Given the potential connection for a heat shock response during the cercaria-schistosomulum transformation and that Hsp70 is widely conserved, we compared the effect of treating cercariae with human skin lipid, linoleic acid, and PES (Fig 2; S2 Video). PES has been shown to prevent Hsp70 from interacting with several Hsp70 client proteins [36]. Experimentally, cercariae respond to a skin lipid smear on the bottom of a petri dish by settling to the bottom of the petri dish and beginning the penetration process [26]. Our observations confirmed this. However, only cercariae located in close proximity to the skin lipid smear seemed to gather at the site where skin lipid was placed; the majority of the cercariae settled to the bottom of the well without regard for the location of the lipid smear. It should be noted that when cercariae were exposed to human skin lipid or linoleic acid (mixed into water and added to cercariae), the cercarial honing response occurred within minutes. We also note that not all cercariae in our 1 mL sample responded to the skin lipid stimulus, as some cercariae could be seen swimming higher in the water column, out of the focal plane (S2 Video); this may correlate with the 60–70% cercarial response previously described in response to human lipids or L-arginine [9].

We next tested the effect of PES, a selective inhibitor of Hsp70. When cercariae were exposed to PES, they initially behaved similarly to the 0.5% DMSO control treatment, whereas the cercariae exposed to skin lipid responded immediately and started settling to the bottom of the well and swimming into or crawling along the surface (Fig 2D, 2G and 2J; S2 Video). However, we were surprised by the result just minutes later. After 5–10 minutes, PES (250 μM)-treated cercariae began to swim to the bottom of the culture plate well, eventually losing their tails to transform into schistosomula (Fig 2J, 2K and 2L). We observed the same effect with a lower treatment concentration (50 μM) of PES but at a later time point (S4 Video). While the majority of the cercariae treated with human skin lipid or linoleic acid honed downward, we observed that 100% of the PES-treated cercariae settled to the bottom of the well and began the penetration behavior (S2 Video). When we co-treated cercariae with PES and skin lipid, the cercariae responded with an effect similar to that of PES alone: all of the cercariae were present at the bottom of the well (S3 Video).

We observed that after exposure to skin lipid (9 minutes) or PES (51 minutes), the cercariae formed clusters (Fig 2H and 2K); this effect was not seen in the 0.5% DMSO control treatment (57 minutes, Fig 2E; S2 Video). Cercariae under PES treatment had not yet formed these clusters at 20 minutes (S4 Video). A majority of the cercariae lost their tails by 1–3 hours in the PES treatment and 1 hour in the skin lipid treatment (Fig 2L and 2I; S2 and S4 Videos); again, this effect was not seen in the 0.5% DMSO control treatment (1 hour 56 minutes, Fig 2F; S4 Video). Within 3 hours, both PES and skin lipid-treated cercariae transformed into schistosomula. For PES-treated cercariae, it should be noted that this honing and transformation occurred in the absence of any host signaling molecules.

Transformation involves several events, notably the loss of tails and loss of water tolerance. The flat appearance of the heads of the cercariae in the skin lipid- and linoleic acid-treated sample at 2 hours indicates the loss of water tolerance and lysis, and further progression in the transformation to the schistosomulum stage, as compared with the corresponding PES-treated sample, in which the heads have a round appearance and are motile (S2 Video). We should also note that the timing for all events seemed to vary somewhat, albeit consistently between cercarial sheds. For example, in one cercarial shed, honing with skin lipids may begin within a minute, in another 3 minutes.

### Treatment of cercariae with other Hsp70 modulators

**Fig 3:**
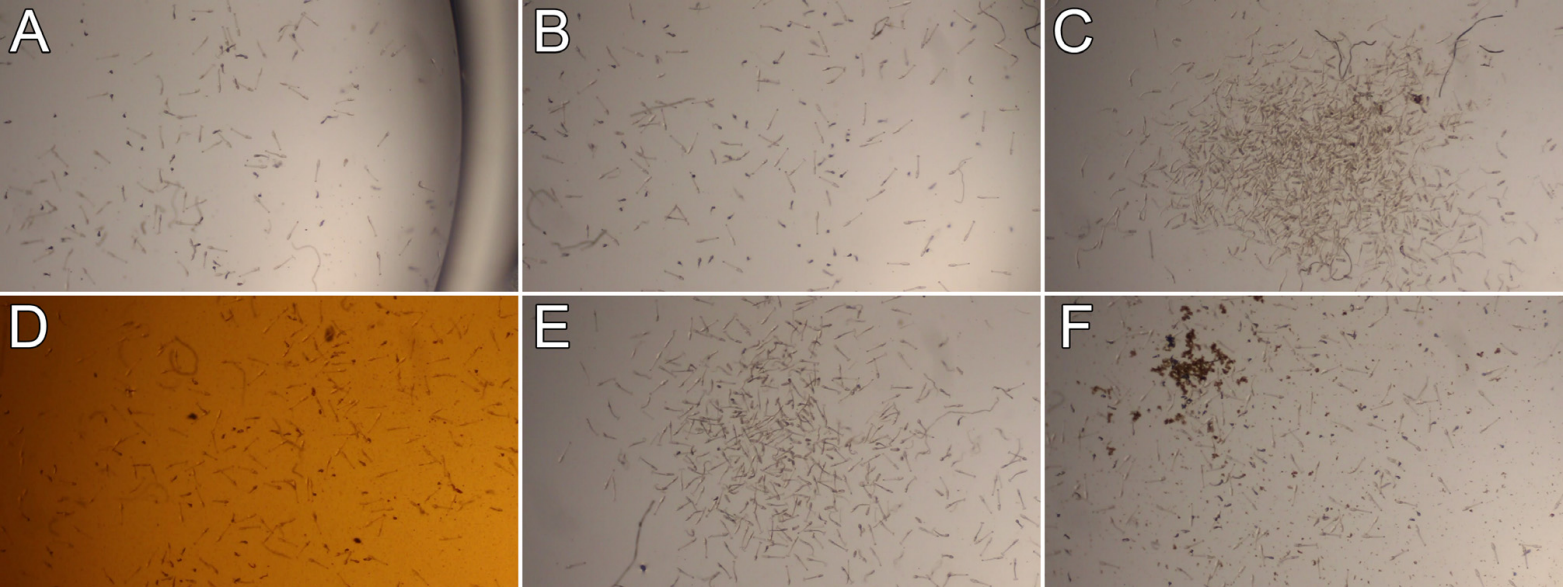
Cercariae treated with other Hsp70 inhibitors do not hone. Cercariae were treated with filtered water (A), 1% DMSO (B), 250 μM PES (C), 500 μM MKT-077 (D), 400 μM 115-7c (E), or 100 μM VER-155008 (F); observed at 2 hours. Each treatment used about 1,000 cercariae in a volume of 1 mL in a 12-well plate well (10× view).

To further determine whether the effect of PES is specific to Hsp70, we treated cercariae with several different Hsp70 modulators, including MKT-077, 115-7c, and VER-155008. MKT-077 functions as an allosteric inhibitor of Hsp70, binding within the nucleotide binding domain of Hsp70 next to its ATP/ADP binding pocket and inhibiting ATP turnover rate. MKT-077 is a rhodacyanine dye originally identified as an anti-tumor agent, and it has been shown to bind mortalin, an Hsp70 family member, and disrupt its interaction with p53 [43]. However, we found no obvious change in behavior of the cercariae in our MKT-077 treatments at 100, 250, or 500 μM concentrations (Fig 3D), with the exception of increased death at 22 hours (S5 Video). Note that the mechanism of action of MKT-077 differs from that of PES, which binds to the Hsp70 substrate binding domain and competitively blocks protein-protein interactions of Hsp70 and its client proteins.

While most pharmacological agents target and inhibit the function of proteins, 115-7c has the unusual property of acting as an activator of Hsp70 protein folding function, leading to an enhanced rate of substrate refolding [44]. It binds to Hsp70 and promotes complex formation between Hsp70 and Hsp40. In our treatments of cercariae with 115-7c, we observed the induction of honing behavior by 2 hours, especially in the 400 μM treatment (Fig 3E); by 22 hours, a majority of the cercariae had lost their tails (S6 Video).

VER-155008 at the concentration used (100 μM) is insoluble in water, and it did not change the behavior of the cercariae (Fig 3F; S7 Video). While there are numerous inhibitors of Hsp70, most utilize a similar mechanism of action. For example, all of the following Hsp70 modulators inhibit Hsp70 nucleotide binding activity or ATPase activity: apoptozole, JG-98, methylene blue, MKT-077, VER-155008, YM-01, and YM-08 (stressmarq.com).

### Treatment of cercariae with Hsp90 inhibitors

**Fig 4:**
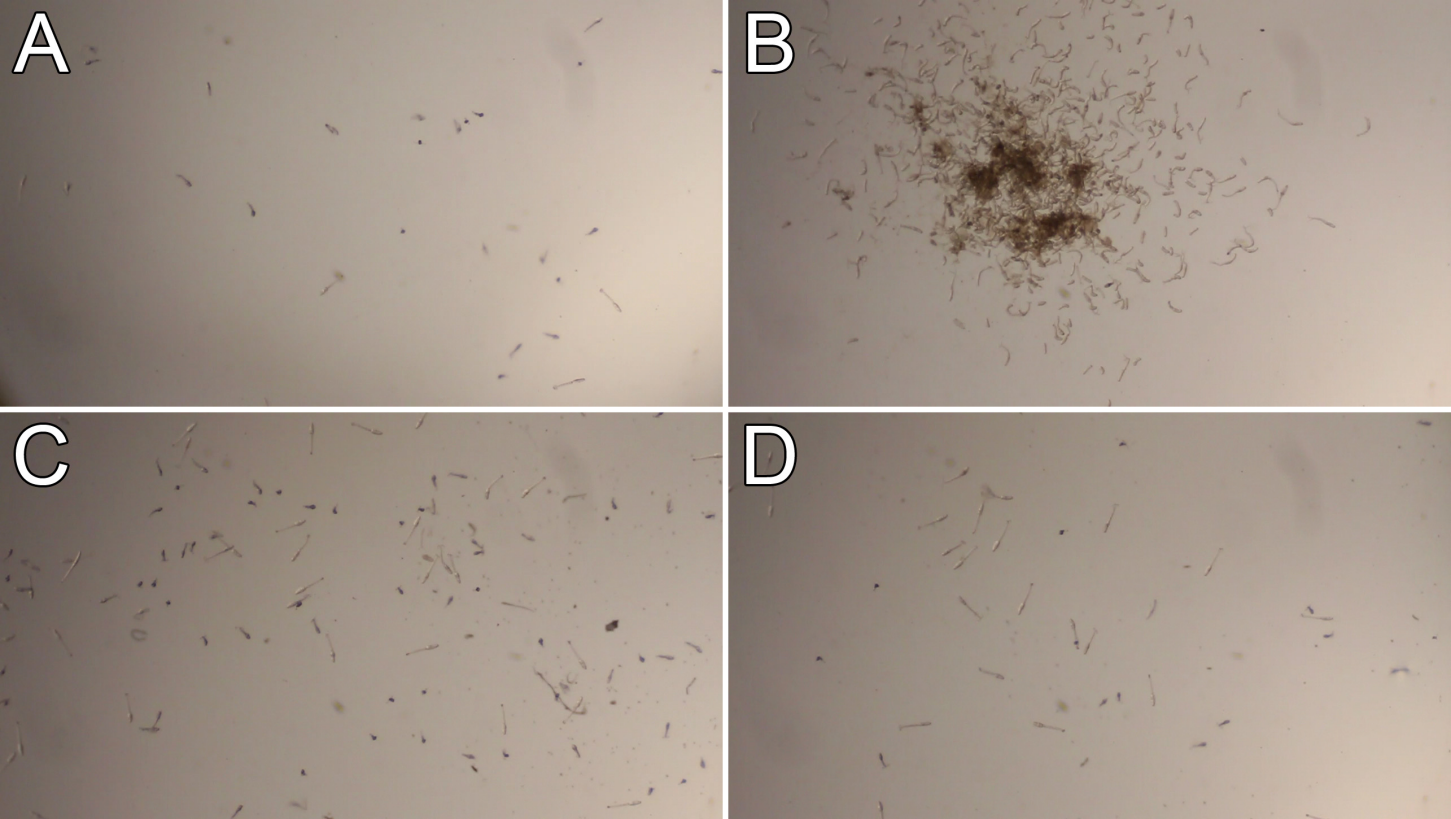
Cercariae treated with Hsp90 inhibitors do not hone. Cercariae were treated with 1% DMSO (A), 250 μM PES (B), 100 μM geldanamycin (C), or 50 μM 17-DMAG (D); observed at 2 hours. Each treatment used about 1,000 cercariae in a volume of 1 mL in a 12-well plate well (10× view).

Since Hsp70 can work with other HSPs as a major effector of the heat shock response pathway, we asked whether another highly conserved HSP, Hsp90, could be involved. We treated cercariae with the Hsp90 inhibitors geldanamycin and 17-DMAG, a water-soluble derivative of geldanamycin. However, treatment with these compounds did not produce a change in cercarial behavior; the cercariae resembled those treated with 1% DMSO (Fig 4; S8 Video).

### Treatment of cercariae with other compounds

Although PES is a potent inhibitor of Hsp70, it was initially described in a screen to identify molecules that block p53-dependent transcriptional activation and apoptosis [54, 55]. PES can also block cisplatin-induced p53 interaction with mitochondrial Bak, a pro-apoptotic molecule responsible for the permeabilization of the mitochondrial membrane, and which thereby blocks p53-dependent activation of apoptosis-associated caspases 8 and 3 [56]. However, it is thought that PES inhibition of p53 acts by inhibition of Hsp70, as PES does not directly interact with p53, BAK, BCL-xL, Grp78, Hsc70, or Hsp90 [36]. The molecular targets or mechanism for p53 regulation of apoptosis is unclear. To determine whether the apoptosis pathway is involved in the honing behavior of cercariae, we blocked caspase activity by treating cercariae with a pan-caspase inhibitor, Z-VAD-FMK. When cercariae were treated with Z-VAD-FMK, we found no change in cercarial honing behavior. Co-treatment with Z-VAD-FMK and PES resulted in a honing behavior similar to that of PES treatment alone (S9 Video).

As an additional treatment, we included praziquantel, the long-standing drug treatment for human schistosome infection. The efficacy of praziquantel treatment depends on the parasite stage for schistosomes; notably, while it can kill cercaria and adult stage schistosomes, it cannot kill the intermediate schistosomulum stage schistosomes [48, 57]. Our treatment of cercariae with 300 nM praziquantel resulted in settling, similar to honing behavior; however, at 24 hours, we observed that while most of the cercariae had died, very few had lost their tails, in contrast to the PES treatment, which resulted in tail loss (in addition to death) for nearly all of the cercariae (S10 Video).

**Fig 5:**
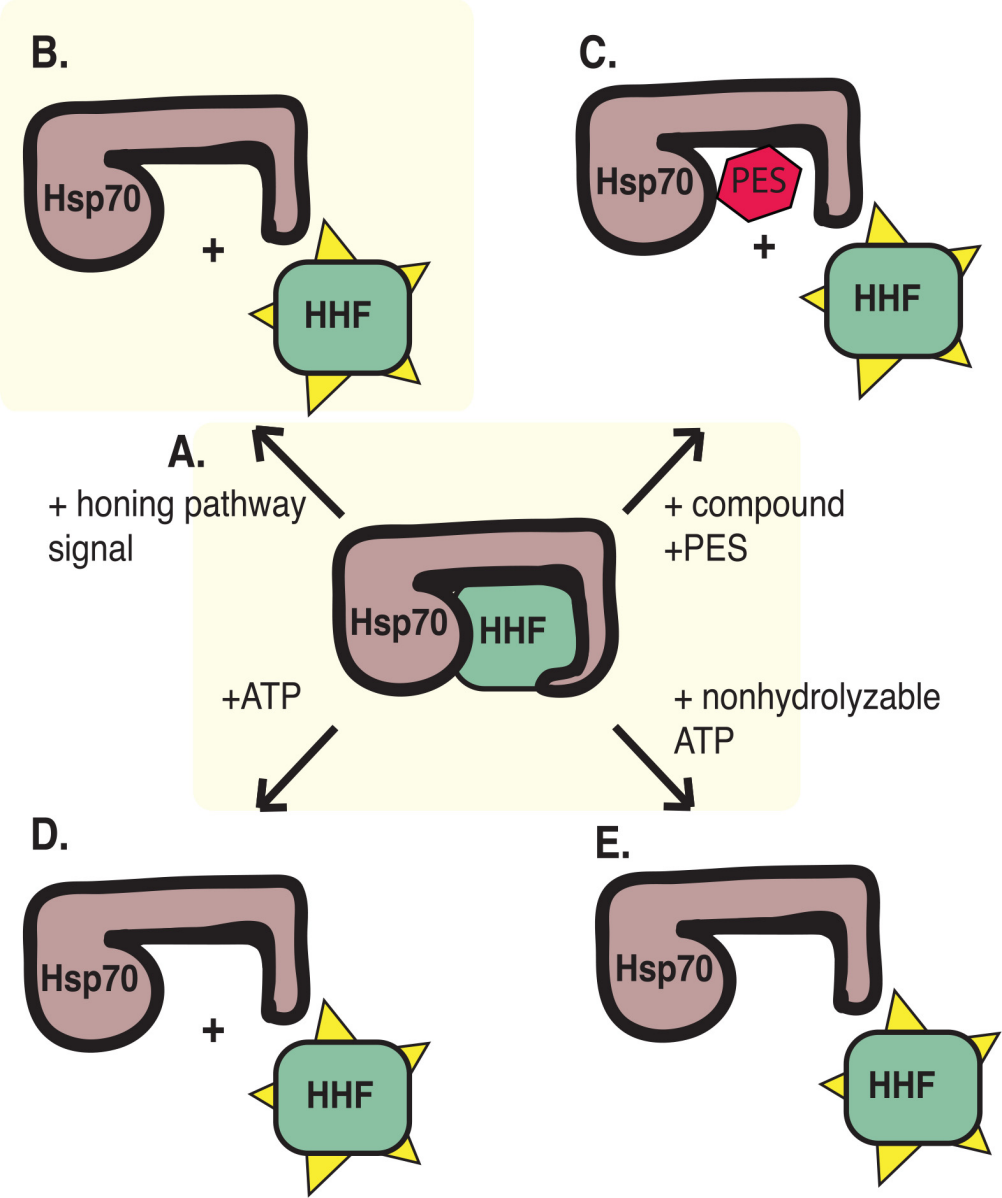
Predictive model of a role for Hsp70 in cercarial honing. (A) In the absence of strong host signals, Hsp70 binds tightly to its client protein, HHF, inhibiting its activity. (B) Host signals are transmitted through a cercarial signal transduction pathway, releasing Hsp70 inhibition of HHF, which functions in cercarial honing. (C) The inhibitor PES blocks Hsp70 activity by binding to the Hsp70 substrate binding domain and releasing Hsp70 inhibition of HHF, resulting in cercarial honing. (D) Addition of 10 mM ATP leads to release of HHF, possibly by binding to the Hsp70 ATPase domain and reducing its affinity for HHF, resulting in cercarial honing. (E) Addition of a non-hydrolyzable form of ATP leads to release of HHF, possibly by preventing ATP hydrolysis and maintaining the weak affinity state of Hsp70 for binding client proteins, resulting in cercarial honing.

**Fig 6:**
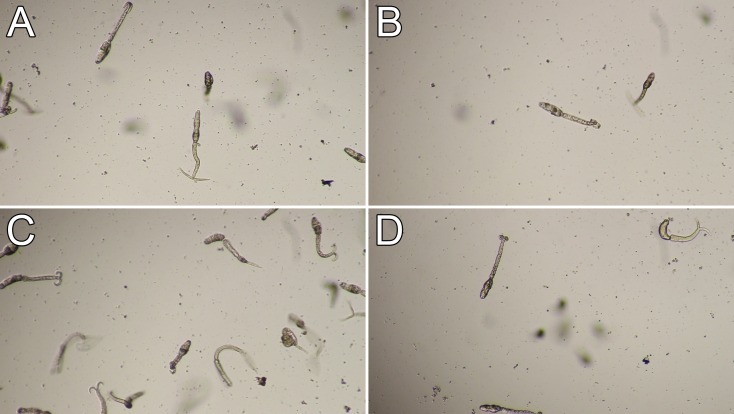
Cercariae treated with ATP, AMP-PNP, and ADP. Cercariae were treated with filtered water (A), 5 mM ATP (B), 5 mM AMP-PNP (C), or 5 mM ADP (D), and observed 2 hours 30 minutes after treatment. Each treatment used about 500 cercariae in a volume of 0.5 mL in a 24-well plate well (40× view).

Functional roles for Hsp70 in the regulation of signal transduction through the binding of client proteins have been recently described and correlate with its intrinsic ATPase activity [29]. When Hsp70 is in an ADP bound state (Hsp70*ADP), Hsp70 interacts with its client protein stably and the Hsp70 lid is in a “closed” state, preventing release of the client protein. When in the ATP bound state (Hsp70*ATP), the Hsp70 lid is opened, allowing the release of the client protein and increasing the on/off rate at the substrate interaction domain [58]. We propose in the regulation of cercarial honing that Hsp70 binds a client protein and functionally inhibits the client protein’s ability to initiate cercarial honing (Fig 5). In accordance with this, if Hsp70 is critical to honing, then we predict that increasing the ATP concentration should cause the Hsp70 lid to open, leading to the release of the client protein and consequentially result in cercarial honing (Fig 5). To test this, we treated cercariae with ATP, AMP-PNP (a non-hydrolyzable ATP analog), and ADP, each at a concentration of 5 mM (intracellular ATP concentration in mammalian cells has been suspected to occur in the millimolar range [59]). While ATP and ADP treatments at this concentration did not show any difference compared to the water alone control treatment, AMP-PNP induced honing behavior within 2 hours 30 minutes (Fig 6; S11 Video).

## Discussion

Understanding the requirements for schistosome infection at the parasite-host interface can expedite the identification of novel targets for prevention of infection or the elimination of newly established infections. This has been observed using a topical skin treatment with inhibitors of the schistosome proteases used by the larval cercarial form, during invasion [60, 61]. The process by which cercariae invade a mammalian host has been well described, but molecular requirements regulating this process are unknown. We present evidence that Hsp70 is involved in the process of cercarial honing and plays a role in a signal transduction pathway to regulate cercarial invasion behavior. Cercariae are released from their molluscan host and have less than 24 hours to find a mammalian host before depletion of their glycogen stores in their tail and body prevents their ability to penetrate host skin [4]. In search of a host, cercariae are distributed in the water column with minimal up and down motion, presumably lying in wait in what might be described as a “still hunting” mode. Cercariae swim randomly in response to water turbulence, light and shadows, and it is thought that they swim toward their host through gradients of body heat and skin chemicals, including linoleic acid, human skin lipid, and L-arginine, with the latter two being the most directionally significant [8, 12, 62]. This chemotactic process is modeled by placing cercariae in water and exposing them to a surface streaked with skin lipid as stimulus. We observed limited chemotaxis in our treatments of cercariae with skin lipid, such that only the cercariae in close proximity to the site where the skin lipid was placed made contact with the lipid. This suggests that cercariae do not swim toward the host over long distances, but lie in wait for a host that comes into close proximity and swim more actively to increase the chance of making contact with the host.

Pharmacological targeting is one way to dissect the molecular pathways that may be involved in cercarial honing. Here, we used a selection of chemical compounds to query a role for Hsp70, Hsp90, and apoptosis in this honing behavior. Based on our observations, we propose that a heat shock pathway is specifically involved in cercarial honing for host invasion. HSPs have been identified in cercarial gland secretions [26, 63] and are among the highest abundance transcripts identified in newly transformed schistosomula [3]. In fact, HSPs have been correlated with cercarial transformation since the late 1980s [64]. However, the role of HSPs has traditionally been connected with the stress response (for review, [22–24]), correlating with the transition from cercaria to schistosomulum, which involves a temperature change from that of ambient water to 37°C host body temperature. Recent evidence in other systems has suggested that HSPs have more diverse functions outside of stress response, including roles in oogenesis and development, lifespan extension, regulation of cancer, fertility and viability [65–71]. In the schistosome molluscan host *B*. *glabrata*, the snail heat shock response is necessary for snail susceptibility to infection, such that a reduced heat shock response in the snail results in resistance to schistosome infection [27]; this suggests an important function in host HSP level for schistosome host invasion.

Our observation that cercariae treated with PES undergo a behavioral change is novel, and it allows for the initial identification of molecular components involved in cercarial honing. Honing occurs in response to skin lipid [72]; however, since cercariae treated with the Hsp70 inhibitor PES show a similar behavior to cercariae treated with skin lipid, we predicted that Hsp70 plays a regulatory role in the signaling required for the honing behavior. Honing induced by PES is concentration-dependent with time, such that lower concentrations require more time for induction to occur (S4 Video). Our treatment of cercariae with two other Hsp70 modulators, MKT-077 and 115-7c, resulted in different behaviors. MKT-077 treatment resulted in a lack of honing, similar to the water control treatment, while treatment with the Hsp70 activator 115-7c resulted in honing behavior, similar to the PES treatment. We propose that while all three Hsp70 modulators tested in this study bind to Hsp70, only PES and 115-7c actively promote the release of its client protein, which can then function to initiate cercarial honing.

Treatment with ATP and its non-hydrolyzable analog, AMP-PNP, could also cause Hsp70 to release its client protein by skewing the Hsp70*ATP/ADP binding state distribution toward Hsp70*ATP (the state at which Hsp70 has low affinity for client proteins). Specifically, we model that in the uninduced honing state (absence of lipid stimulus), Hsp70 interacts with and negatively regulates or inhibits the function of an Hsp70 client protein, which we call Hsp70 Honing Factor (HHF). Upon upstream signal activation by skin lipid, Hsp70 releases HHF, which allows the activation of further signaling to trigger the honing behavior (Fig 5). This model is not in disagreement with current models describing a function for Hsp70 signaling [29].

A signaling pathway required to induce cercarial honing implies that many potential signaling factors could be involved, beginning from the receptor(s) that senses skin lipid, potential kinases or phosphatases, through Hsp70, HHF and its targets. We reasoned that other HSPs, such as Hsp90, could be involved, as Hsp90 is reported to interact with client proteins in signaling as well [73]. However, in our treatment of cercariae with Hsp90 inhibitors geldanamycin and 17-DMAG, a geldanamycin derivative, we did not observe any obvious change in the behavior of the cercariae. Next, we considered the potential involvement of apoptosis in honing induction. PES can block cisplatin-induced p53 activation of apoptosis [36]. Our treatment with the apoptosis inhibitor Z-VAD-FMK also did not result in any obvious change in the behavior of the cercariae. Interestingly, praziquantel treatment led to honing behavior similar to that resulting from PES treatment; however, at 24 hours, most of the cercariae had not lost their tails, indicating that transformation did not occur. In contrast, tail loss occurred for the skin lipid, linoleic acid, PES, and 115-7c treatments by 24 hours (S2 Video). This observation leads us to speculate that cercarial honing involves specific signaling to cause the loss of tails (transformation) in addition to a change in the swimming pattern (settling).

Further effort will be necessary to identify the signaling components involved in cercarial honing, including the proposed Hsp70 client protein, HHF, and to better understand the relatively recently described role of Hsp70 in signaling [29]. Under ideal circumstances, genetics approaches such as gene knock-downs and knock-outs would be appropriate to identify honing components. However, these tools have not been thoroughly developed for use in developing or mature cercariae. Our group and others are working on developing methods to overcome these technical challenges [74–79]. Analysis in cercariae is challenging, as cercariae are short lived, transient, and the necessary proteins for swimming and host invasion have already been produced prior to exit from the snail host. Genetic manipulations of early developing cercariae within sporocysts may be possible, but in the case of Hsp70 and potentially other proteins, knock-down or knock-out could result in the loss of viability or production, not because of protein targeting problems, but because of the multipurpose nature of this particular protein. HSPs are the most abundant proteins expressed in the schistosome egg and miracidium [80]. However, a reduction of the hest shock response in the intermediate snail host, *B*. *glabrata*, makes the snail resistant to schistosome infection [27], suggesting a critical role for the heat shock pathway for intermediate host susceptibility. Consequently, it would not be a far stretch to speculate whether inhibition of miracidial HSPs could affect invasion of the snail host.

In this study, we have just pierced the surface and glimpsed at molecular components that contribute to cercarial honing. We have found no similar observation where Hsp70 signaling affects a whole organism and its behavior directly, leading to stimulating questions such as: how does signaling quickly and directly regulate cercarial behavior, and are there other organisms that are similarly regulated? Additionally, schistosomiasis affects nearly 240 million people globally. Understanding the molecular requirements for cercarial honing and invasion, as well as those for early schistosomulum survival, could identify new potential drug targets and transition schistosome control from treatment to prevention.

## Supporting Information

S1 Fig Phylogenetic tree of Hsp70. Peptide sequences of Hsp70 closely homologous to those of *S*. *mansoni* (NCBI accession CCD76164, labeled *S*. *mansoni* 637 aa; and CCD76236, labeled *S*. *mansoni* 648 aa) were chosen from several species (*A*. *thaliana* 651 aa, NP_195870; *C*. *elegans* 640 aa, NP_503068; *D*. *melanogaster* 641 aa, NP_524063; *D*. *rerio* 643 aa, AAH56709; *D*. *rerio* 650 aa, AAH63946; *E*. *coli* 638 aa, WP_000516131; *H*. *sapiens* 646 aa, NP_006588; *H*. *sapiens* 655 aa, AAI12964; *M*. *musculus* 646 aa, BAE30272; *M*. *musculus* 655 aa, AAH50927; *S*. *cerevisiae* 649 aa, NP_009478; *S*. *haematobium* 648 aa, KGB42118; *S*. *japonicum* 648 aa, AAC00519; *X*. *laevis* 650 aa, NP_001080068; *X*. *laevis* 655 aa, NP_001080064) and aligned using ClustalW2. The phylogenetic output was used to generate the tree using TreeView X software.
(TIF)

S1 Video Cercariae treated with filtered water or DMSO (0.5, 1%) at approximately 10 minutes and 2 hours (40× view). (MP4)

S2 Video Cercariae treated with 0.5% DMSO, human skin lipid, 0.1% linoleic acid, or 250 μM PES at approximately 10 minutes and 1 hour (40× view). (MP4)

S3 Video Cercariae treated with filtered water, human skin lipid, skin lipid / 250 μM PES, or 250 μM PES at approximately 10 minutes and 1 hour (40× view). (MP4)

S4 Video Cercariae treated with 0.5% DMSO or PES (50, 150, 250 μM) at approximately 2 minutes, 10 minutes, 20 minutes, 1 hour, 2 hours, 3 hours, 4 hours, and 22 hours (10× view). (MP4)

S5 Video Cercariae treated with filtered water or MKT-077 (50, 250, 500 μM) at approximately 2 minutes, 10 minutes, 20 minutes, 1 hour, 2 hours, 3 hours, 4 hours, and 22 hours (10× view). (MP4)

S6 Video Cercariae treated with 1% DMSO or 115-7c (100, 200, 400 μM) at approximately 2 minutes, 10 minutes, 20 minutes, 1 hour, 2 hours, 3 hours, 4 hours, and 22 hours (10× view). (MP4)

S7 Video Cercariae treated with filtered water, 0.1% DMSO, or 100 μM VER-155008 at approximately 10 minutes, 30 minutes, and 1 hour (10× view). (MP4)

S8 Video Cercariae treated with filtered water, 1% DMSO, human skin lipid, 0.1% linoleic acid, 100 μM geldanamycin, 50 μM 17-DMAG, 250 μM PES, 500 μM MKT-077, or 400 μM 115-7c at approximately 20 minutes, 40 minutes, 1 hour, 2 hours, 3 hours, 4 hours, and 24 hours (10× view). (MP4)

S9 Video Cercariae treated with 1% DMSO, 25 μM Z-VAD-FMK, 25 μM Z-VAD-FMK / 250 μM PES, or 250 μM PES at approximately 10 minutes, 30 minutes, and 2 hours (40× view). (MP4)

S10 Video Cercariae treated with filtered water, 0.1% ethanol, 300 nM praziquantel, or 250 μM PES at approximately 10 minutes, 4 hours, and 24 hours (10× view). (MP4)

S11 Video Cercariae treated with filtered water, 5 mM ATP, 5 mM AMP-PNP, or 5 mM ADP at approximately 6 minutes, 32 minutes, 1 hour 2 minutes, and 2 hours 32 minutes (40× view). (MP4)
