# Supplementary figures and images for: Hsp70 May Be a Molecular Regulator of Schistosome Host Invasion
Source: PLoS Negl Trop Dis. 2016 Sep 9;10(9):e0004986. doi: 10.1371/journal.pntd.0004986 (PMC5017621; doi:10.1371/journal.pntd.0004986)

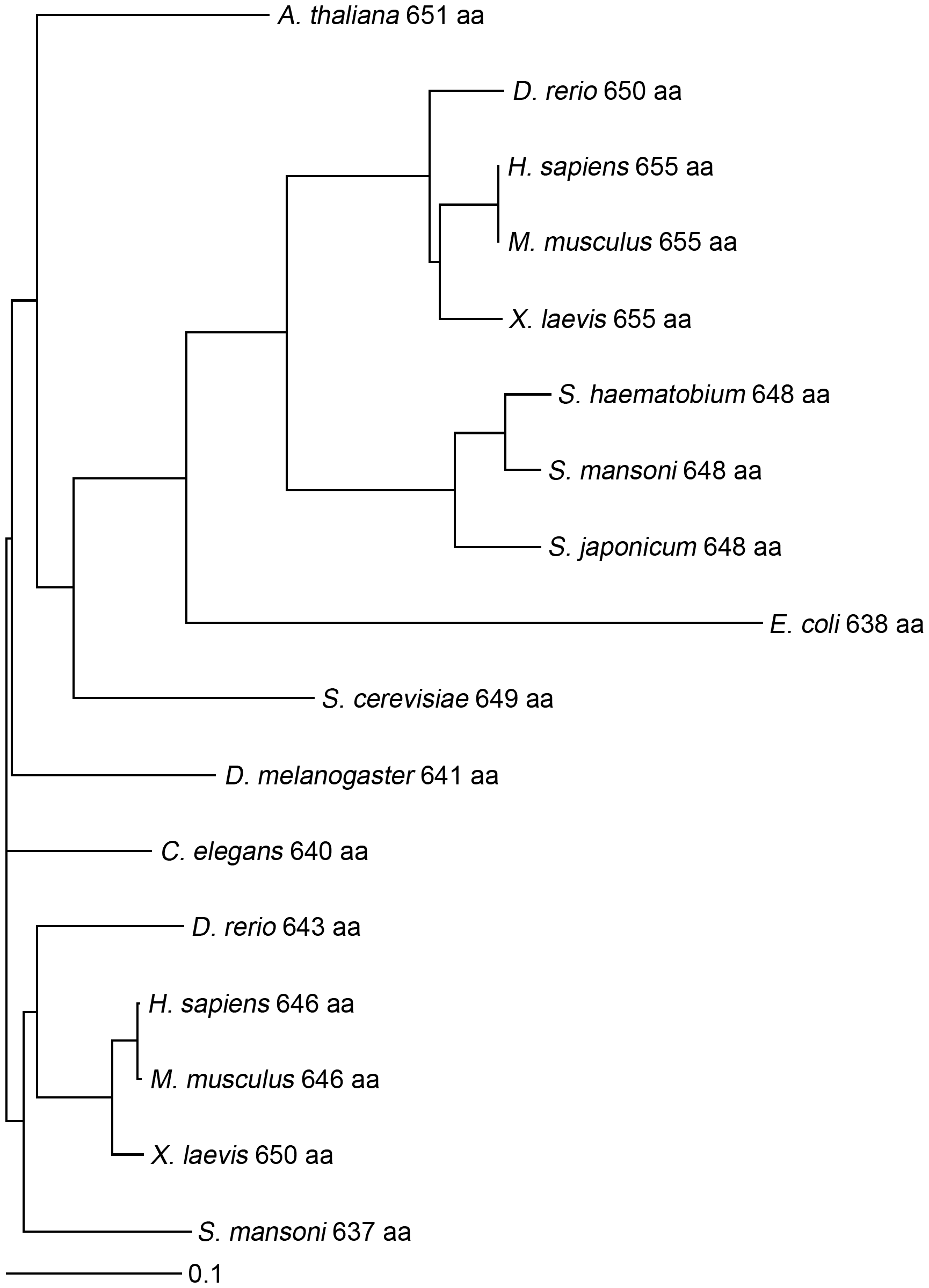

Supplement: S1 Fig — Peptide sequences of Hsp70 closely homologous to those of S. mansoni (NCBI accession CCD76164, labeled S. mansoni 637 aa; and CCD76236, labeled S. mansoni 648 aa) were chosen from several species (A. thaliana 651 aa, NP_195870; C. elegans 640 aa, NP_503068; D. melanogaster 641 aa, NP_524063; D. rerio 643 aa, AAH56709; D. rerio 650 aa, AAH63946; E. coli 638 aa, WP_000516131; H. sapiens 646 aa, NP_006588; H. sapiens 655 aa, AAI12964; M. musculus 646 aa, BAE30272; M. musculus 655 aa, AAH50927; S. cerevisiae 649 aa, NP_009478; S. haematobium 648 aa, KGB42118; S. japonicum 648 aa, AAC00519; X. laevis 650 aa, NP_001080068; X. laevis 655 aa, NP_001080064) and aligned using ClustalW2. The phylogenetic output was used to generate the tree using TreeView X software. (TIF) [file pntd.0004986.s001.tif]
